# Enhancement or suppression: a double-edged sword? Differential association of digital literacy with subjective health of older adult—evidence from China

**DOI:** 10.3389/fpubh.2024.1395162

**Published:** 2024-09-20

**Authors:** Qi-Song Yan, Qiao Guo

**Affiliations:** ^1^School of Management, Chongqing University of Science and Technology, Chongqing, China; ^2^Department of Anesthesiology, The Second Affiliated Hospital of Chongqing Medical University, Chongqing, China

**Keywords:** information literacy, subjective health, older adult, healthy aging, digital health, wearable electronic devices, mediation analysis

## Abstract

**Background:**

The emergence of an aging society and the digital age makes healthy aging a hot topic in Chinese society. This paper explores the associations between digital literacy and the subjective health of older adult individuals in PR China, offering insights that May assist policymakers and service providers in developing strategies and interventions suited to the digital era, potentially enhancing the healthy aging process for this demographic in China.

**Methods:**

This study utilized data from the China Longitudinal Aging Social Survey. Initially, demographic variables of 2086 individuals in the sample were analyzed. Subjective health differences among different populations and correlations between core variables were examined. Subsequently, multivariate linear regression and chain mediation methods were utilized to examine the relationships and potential pathways among the three dimensions of digital literacy and the subjective health of older adult individuals.

**Results:**

(1) The subjective health status of older adult individuals in China was generally favorable, with an average score of 3.406 ± 0.764. (2) There was no direct correlation observed between the frequency of digital information use and the subjective health of the older adult (*b* = −0.032, *p* > 0.1). Digital entertainment information (*b* = 0.294, *p* > 0.1) did not show a significant effect, whereas life management information (*b* = 0.437, *p* < 0.01) demonstrated a positive association. Similarly, the use of smart healthcare devices (*b* = 0.842, *p* < 0.001) indicated a positive association (3) The frequency of digital information use indirectly enhanced the subjective health of the older adult through life management digital information and the use of smart healthcare devices, but had no indirect effect through entertainment and leisure digital information.

**Conclusion:**

Digital literacy is significantly correlated with the subjective health of the older adult, especially when they acquire life management information and utilize smart healthcare devices. However, a potential negative relationship is suggested between digital entertainment information and the subjective health of older adult individuals. Therefore, digital infrastructure should have prioritized the provision of high-quality, age-friendly digital applications for the older adult. This approach could have better harnessed the potential of digitalization to enhance health and well-being in older adults.

## 1 Introduction

With the improvement of public health standards and nutritional conditions in China, the average life expectancy of Chinese people has increased to 78 years. Simultaneously, the number of births in China has sharply declined in recent years, making the rapid aging of the Chinese population an inevitable trend (1). According to data from the National Bureau of Statistics, in 2022, the population aged 65 and above reached 209 million, accounting for 14.9% of China’s total population (2). This figure is expected to continue to grow. Aging is not only a trend in China, but also a global phenomenon. Data from the United Nations’ “World Population Prospects 2022″ showed that the number of people aged 65 and over is continually increasing worldwide. It is projected that this figure will rise from 761 million in 2021 to 1.6 billion by 2050. Therefore, the issue of how to achieve healthy aging for the older adult is increasingly receiving attention from countries around the world (3). Therefore, how to achieve healthy aging for the older adult in China is increasingly receiving attention. To address this long-term challenge, the Chinese government is actively exploring aging models that are suitable for China’s national conditions, while also actively learning from the healthy aging models of other aging societies around the world. Among them, healthy aging empowered by digital information technology has received high attention. China has introduced the Internet for more than 30 years, and the popularity of various information technology applications based on the Internet is high. The older adult can also have wide access to these new digital information application technologies, including digital smart healthcare devices suitable for the older adult and other application technologies are gradually popularized in the market (4, 5). This makes it possible for digital information technology to empower healthy aging for the older adult.

### 1.1 Previous research and its limitations

Digital literacy(DL) is a multidimensional concept that not only encompasses technical skills for handling digital information but also deeper cognitive and social interaction abilities (6). Compared to digital competence, which focuses on information processing and content creation, and digital skills, which focus on the specific abilities required for digital tasks, digital thinking emphasizes innovation and critical thinking in the application of digital technologies. Digital literacy is a broader concept that includes digital cognition, digital thinking, and digital skills. Digital empowerment for the older adult in health and aging care has become a developmental trend in aging societies. Therefore, it is imperative to harness digital technologies judiciously to provide enhanced services and solutions for health and aging care (7). Meanwhile, previous research has revealed two contrasting associations between digital information technology and the health of the older adult: the “enhancement hypothesis” and the “suppression hypothesis.” The enhancement hypothesis posits that digital information and communication technologies empower older adult individuals in managing their health (8, 9), thereby enhancing their self-management capabilities (10), and reshaping their approach to health management (11). Some studies have also found that internet use among the older adult contributes to a sense of belonging and self-esteem (12), alleviates depression (13), and that more frequent internet usage correlates with lower levels of social loneliness (14). DL among the older adult is consistently associated with better psychological health (15, 16), and is correlated with higher levels of life quality and well-being (17). On the other hand, the suppression hypothesis indicates that there May not be a direct association between DL and psychological health. The rapid pace of societal transformation may leave the older adult struggling to adapt, presenting a greater number of life challenges and a negative association with their psychological well-being (18). This is particularly challenging for those with lower socioeconomic status, including the older adult and low-income individuals. Insufficient acquisition of digital skills can exacerbate their health issues (19–22). Furthermore, the use of wearable health devices May induce technological anxiety among the older adult (23, 24), thereby affecting their psychological health.

Previous research has primarily investigated the correlation between internet usage frequency and the mental health of older adults (25), However, these studies have not sufficiently focused on the trend of digital health applications for this demographic, presenting certain limitations. Firstly, the frequency of internet use does not fully represent the DL of the older adult. DL includes not only digital usage skills but also digital cognition and socio-emotional dimensions (26). The European Commission’s definition of digital competence for citizens encompasses five areas: information and data literacy, communication and collaboration, digital content creation, safety, and problem-solving. In summary, DL is a multidimensional concept that includes skills for acquiring, selecting, sharing, and using digital information (27–29). Research on DL should shift from a single dimension of internet use frequency to a multidimensional analytical framework (30, 31). This study evaluates the DL of the older adult from three dimensions: digital information acquisition, digital information selection, and digital usage skills. Digital information acquisition is the foundation of DL, reflecting the basic survival and adaptive capabilities of the older adult in the digital society. Digital information selection, which entails the older adult’s discernment of digital information that meets their needs following cognitive processes such as emotion and critique, reflects their information literacy and critical thinking skills. Digital usage skills, a core component of DL, refer to the proficiency of the older adult in utilizing digital devices and software. Secondly, while previous studies have predominantly addressed the dual “enhancing” and “inhibiting” aspects of internet use in relation to the health of older adults, they have primarily focused on variations in socio-economic status, with less emphasis on the correlation between digital content and the health of older adults (16, 32). The relationship between an individual’s perceived health and the various kinds of digital information they encounter May vary among older adults, possibly because of differences in their abilities to understand and manage a range of digital content (19, 33). Thirdly, much of the past research has discussed the correlation between DL and the mental health of older adults, but there is a lack of research examining its relationship with the overall health of older adults, which encompasses both physical and mental health (34). Subjective health (SH), which integrates various personal and societal factors in the evaluation of health conditions, provides a more accurate reflection of an individual’s overall health status (35, 36). SH is closely associated with mortality risk, and its predictive capacity for happiness and quality of life surpasses that of mental and physical health, and is also more precise than professional medical evaluations (37). Therefore, it is more appropriate to utilize the SH of older adults as a measure, rather than focusing exclusively on mental health.

### 1.2 Formulation of research questions

**Figure 1:**
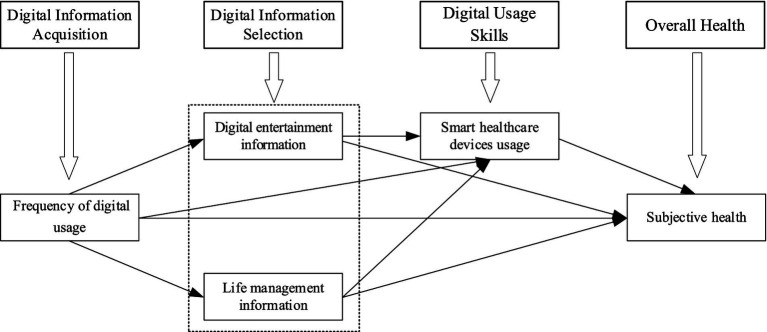
Analysis framework diagram.

Upon reviewing previous research, this study identifies a critical issue that merits further refinement and exploration: examining the relationship between multidimensional DL and the SH of older adults from a multifaceted perspective. To address this issue, under the framework of multi-dimensional DL, the DL of the older adult is subdivided into three dimensions: digital information acquisition, digital information selection, and digital usage skills, among which digital information acquisition, is a prerequisite for the formation of DL in the older adult (38). These three dimensions are operationalized as three variables: the frequence of digital usage (FDU), digital information content, which includes life management information (LMI) and digital entertainment information (DEI), and the smart healthcare devices usage (SHDU). On this basis, it analyzes how the three dimensions of DL, which have a progressive relationship, jointly act on the SH of the older adult (Figure 1). The innovation of this study is the identification of digital information selection as a key dimension of DL. It explores how the demand for different types of digital information content is satisfied to enhance the SH experiences of older adults, revealing their diversity. In the context of the increasingly obvious trend of digitalization in Chinese society and societies around the world, and the increasingly prominent issue of healthy aging, this research helps to understand the relationship and mechanism between DL and healthy aging of the older adult from a multi-dimensional perspective more comprehensively, and provides a theoretical basis for optimizing DL and healthy aging services for the older adult at the policy level globally.

## 2 Literature review and research hypothesis

### 2.1 Frequency of digital usage and subjective health

The Internet, as a modern communication tool, exhibits a significant correlation with the psychological well-being and social engagement of older adult. The most basic function of the Internet is to facilitate interpersonal information contact and emotional exchange, helping people to gain social support. When the population mobility of a society increases, using the Internet can help the older adult contact their children who are not at home, which can reduce the loneliness of the older adult (39). Observations suggest that among older adults, long-term internet use is often concurrent with reports of physical and mental well-being, as well as with lower incidences of functional disabilities (40). For those older adult people with less social interaction, using the Internet and other digital technologies can help alleviate symptoms of depression (25, 41). In addition, access to the Internet can not only help the older adult maintain their social circle but also enhance the social network support of the older adult (17). This can broaden their social channels and facilitate their social interaction capabilities, potentially leading to an enhancement in the quality of their social interaction and social support (42). The older adult can expand their social capital through information and communication technology, which means that they can get more social support from digital media, and the pleasure and SH they get from life will be enhanced accordingly (43). Especially, there is a positive correlation between the degree of DL improvement and the sense of happiness among low-income groups (44). In summary, the increased FDU by the older adult May reduce depression and enhance happiness, and self-health assessment (45, 46). Based on this, the first hypothesis of this paper is derived.

Hypothesis 1: There is a positive correlation between the frequency of digital usage and the subjective health of older adults. The higher the frequency of digital usage, the higher the level of their subjective health.

### 2.2 The mediating effect of digital information content

Most previous studies have suggested that older adult individuals’ use of the internet for social interaction and information acquisition helps them gain support from family and society (17, 47), enhance their social cognition (48), and contributes to their subjective evaluation of their own health. However, the types of internet information accessed by the older adult are diverse. Although previous research has categorized the internet information accessed by the older adult into entertainment and leisure, life management, etc. (49, 50), there has been little in-depth comparison of the relationship between different types of internet information and the health of the older adult. The preference for digital information selection is a reflection of DL (51), as the older adult choose different types of digital information based on their varying needs and motivations (11), which May relate differently to their health.

There are two main viewpoints regarding the relationship between DEI and the health of the older adult: positive promotion and negative inhibition. The first viewpoint observes a correlation between light entertainment and leisure activities and the mental health of individuals (52), with online entertainment and leisure activities helping to restore attention (53), positively affecting self-cognitive abilities (54), and improving health indicators such as physical balance and processing speed (50, 55, 56). However, recent studies have found negative inhibition, suggesting that excessive use of entertainment digital technology, such as addiction to DEI, can pose health risks to the older adult. Long-term use of electronic devices is detrimental to their eyesight, causing discomfort or eye diseases (57). The degree of digital addiction negatively affects sleep quality, with the more digitally addicted older adult spending more time on social media, leading to poorer sleep quality (58). Reducing the use of DEI can lower levels of depression, anxiety, and stress (59).

Previous research has also examined the relationship between digital information and the daily life of the older adult. Studies have found that timely access to information on health management, shopping, transportation, and weather forecasts helps them manage their daily routines and expectations of life (32, 60). The comprehensiveness and diversity of digital information meet the diverse life management needs of the older adult. LMI is closely related to the daily lives of the older adult, helping to enhance their sense of control over life and boost their confidence and self-esteem, thereby promoting their SH. Such information is typically correlated with positive SH in the older adult (42). Digital participation is often associated with various aspects of social support, psychological well-being, and self-efficacy of the older adult, enhancing their quality of life and life satisfaction (61, 62). Health management is an important part of the older adult’s life, and when they focus on health information, they actively acquire health information from online health information systems. Corresponding smart health systems support the older adult in acquiring, retrieving, and providing feedback on health information through human-computer interaction (63), making it easier for them to obtain health-related life information and promoting independent healthy living (64).

The relationship between digital information and the health of older adults is complex and multifaceted. On one hand, DEI May enhance the older adult’s self-cognition, but excessive indulgence can harm their mental health. On the other hand, life management digital information is often positively associated with various health outcomes among the older adult. In summary, DEI and life management digital information have different relationships between FDU and SH. Thus, the second hypothesis of this paper is derived:

Hypothesis 2a: Digital entertainment information has a negative mediating effect between the frequency of digital usage and subjective health.

Hypothesis 2b: Life management information has a positive mediating effect between the frequency of digital usage and subjective health.

### 2.3 The mediating effect of smart healthcare device usage

The advent of digitization has led to the development and proliferation of smart healthcare devices, and the ownership and use of such devices by the older adult is an important dimension of DL. Previous research has found that the use of smart medical devices for electronic health monitoring can improve the health of the older adult (8). Smart wearable devices and immersive virtual reality devices suitable for the older adult can assist the older adult in obtaining health information, provide medical care monitoring, and help the older adult better manage their lives and health conditions (65, 66). These devices assist the older adult in their daily lives, meet the humanistic needs of the older adult with illnesses, improve their quality of life and daily functions (9, 67), enhance the confidence of the older adult in independent living (68), and improve the experience of the older adult in medical care (69). The process of the older adult SHDU can also enhance their understanding and participation in health issues (70), promote social interaction among the older adult about health care device information (71), and enhance their awareness of health communication. In addition, research has also found that smart healthcare devices have potential application value in coping with stress management (72), can have a positive impact on the physical and psychological health of the older adult, and improve their health management and quality of life (73). In general, through the use of digital information, it can promote the use of smart and digital health devices by the older adult, and have a positive significance for the health management and health perception of the older adult (74, 75). Based on this, the third hypothesis of this paper is derived.

Hypothesis 3: The level of the smart healthcare devices usage has a positive mediating effect between the frequency of digital usage and subjective health.

### 2.4 The chained mediating effect of digital information content and SHDU

The choice of different digital information content by the older adult has different empowerment effects on their use of digital health care devices. Older adult people who pay more attention to LMI can access more digital life device information (76), can communicate with peers who have common needs about digital health care devices, and can acquire more digital health device usage skills (8). Moreover, it can also indirectly promote them to participate better in digital social activities (70), thereby improving the quality of their social interaction and social support (42), and then help the older adult to manage their health better (77). However, older adult individuals who focus on entertainment and leisure digital information, while still engaging in social interactions, tend to concentrate on entertainment topics rather than topics related to digital technology operation. These older adult individuals May face challenges such as insufficient skills and incomplete information when using digital healthcare devices. Although these challenges are not universal, they can exacerbate their anxiety towards digital technology and other psychological disorders (78, 79). Compared to older adult individuals who focus onLMI, those who concentrate on entertainment and leisure digital information might be less capable of using smart healthcare devices, leading to a lack of confidence in their own health due to insufficient skills in operating these devices. Combining the hypotheses on the relationship between digital information content and SH, as well as the relationship between the use of smart healthcare devices and SH among the older adult, we can propose the fourth hypothesis of this paper.

Hypothesis 4a: Between the frequency of digital usage and subjective health, digital entertainment information and the smart healthcare devices usage have a negative chain mediating effect.

Hypothesis 4b: Between the frequency of digital usage and subjective health, life management information and the smart healthcare devices usage have a positive chain mediating effect.

## 3 Methods

### 3.1 Data

This study utilized data from the China Longitudinal Aging Social Survey (CLASS), a nationwide survey conducted by Renmin University of China (RUC) on the mainland of China. The project systematically and regularly collected data on basic personal information, health and services, and socioeconomic status of Chinese older adults aged 60 and above (80). The survey sample covered 28 provinces in mainland China. The academic community highly recognized the quality, representativeness, and credibility of the survey data, and many scholars used this data to study the health status of Chinese older adults and its influencing factors. The survey of this research was reviewed and approved by the Scientific Research Ethics Committee at Renmin University of China, and all respondents signed a written informed consent (81). CLASS has not conducted any new surveys since 2019, and this paper used the survey data from 2018 as the analysis sample. The CLASS 2018 survey collected data from a total of 11,419 individuals aged 60 and above. This paper utilizes demographic variables from this survey, such as age and gender, as well as data on SH, FDU, digital information content, and the SHDU. Since the study of DL in the older adult is predicated on their engagement with the internet, this research focused solely on older adult individuals who have a history of internet use, resulting in a final dataset comprising 2,086 case studies.

### 3.2 Variables and measurement

#### 3.2.1 Dependent variable

The dependent variable in this paper is SH. SH is an individual’s overall assessment of their own health status. It integrates various personal and social factors in the evaluation of health status, reflecting the overall health status of an individual (35, 36). CLASS 2018 used two questions to measure this variable. (a) How do you feel about your current physical health status? The options used a five-point Likert scale, ranging from “very unhealthy” to “very healthy,” coded as 1–5, respectively. (b) Compared with people of the same age, how do you feel about your health status? The options also used a five-point Likert scale, ranging from “much worse” to “much better,” coded as 1–5, respectively. The Cronbach’s alpha for the two items is 0.8166, indicating high reliability. Factor analysis was used to combine the two items into a SH factor. The higher the factor score, the healthier the respondents subjectively believed they were.

#### 3.2.2 Independent variables

The independent variable in this study is DL, which specifically includes three dimensions: digital information acquisition, digital information selection, and digital usage skills. Digital information acquisition is fundamental to DL, mirroring the basic survival and adaptability of the older adult in a digital society. Therefore, this study operationalizes “digital information acquisition” as “the frequency of digital information usage.” The frequency of usage reflects the extent of digital information intake and output among older adults. Consequently, the frequency of digital information usage is posited as a measure of the older adult’s proficiency in obtaining digital information. CLASS2018 measured the internet usage of Chinese older adult individuals using three questions: “Do you use the internet?,” “How frequently have you used the internet in the past 3 months?,” and “How frequently have you used customized messages on your mobile phone in the past 3 months?.” The options ranged from “never” to “always,” coded from 1 to 5, respectively. The Cronbach’s alpha for the three items is 0.7901, indicating relatively high reliability. Factor analysis was used to combine the three items into a digital information usage frequency factor. The higher the factor score, the higher the frequency of digital information use among the respondents.

The ability to select digital information refers to the capacity of the older adult to filter and adopt digital information that aligns with their personal needs and interests within a digital environment, through the application of critical thinking and emotional judgment. This process demands that older adults possess advanced cognitive assessment skills to discern and select content that is relevant and valuable from Internet platforms. The cultivation and enhancement of digital information selection skills are indispensable components in the construction of DL for the older adult, as they are directly linked to their advanced cognitive functions of information identification, evaluation, and decision-making. The questionnaire asked respondents, “What do you generally do online?” This multiple-choice question included options such as “text chatting,” “reading news,” “listening to music, watching videos,” as well as “shopping,” “transportation and travel,” “health management,” and “investment and financial management.” If the respondent selected an option, it was coded as 1; otherwise, it was coded as 0. The first three items primarily involved entertainment and leisure content, while the latter four items involved life management content. Item response theory models were used to fit the first three items into a “digital entertainment information” index and the latter four items into a “life management information” index. Higher scores on these indices indicate a greater likelihood of the respondents using internet information for entertainment and leisure or life management, respectively.

Digital usage skills refer to the ability to use and operate digital technologies, platforms, and tools (82). The use of smart healthcare devices by the older adult to monitor their health status reflects their digital skills. Therefore, this study operationalizes “digital usage skills “as “the use of smart healthcare devices.” The questionnaire asked respondents, “Have you used any of the following smart devices?” This multiple-choice question included options such as “smart wheelchair,” “smart wristband,” and “smart sleep monitor.” If the respondent selected an option, it was coded as 1; otherwise, it was coded as 0. Item response theory models were used to fit the three items into a “use of smart healthcare devices” index. A higher index score indicates a higher level of use of smart healthcare devices by the respondents.

It should be noted that in the multivariate regression analysis stage, all three dimensions of DL were used as independent variables to analyze the relationship between the three dimensions and the SH of the older adult. In the mediation analysis stage, the FDU was used as the independent variable, and digital information content and the SHDU were used as mediating variables to analyze the correlation mechanism between the different dimensions of DL and SH among the older adult.

#### 3.2.3 Control variables

The overall health status of older adults is associated with a multitude of factors, among which variables such as gender, age, and socio-economic status are common influencing factors (83, 84). Therefore, this paper uses variables that reflect demographic characteristics and socio-economic status, such as gender, age, years of education, marital status, place of residence, and individual annual income, as control variables. The gender variable was binary, with males coded as 1 and females coded as 0. Age was a continuous variable, with the ages of the older adult respondents in this study ranging from 60 to 108 years old. Years of education was a continuous variable, with no schooling, primary school, junior high school, high school, junior college, and undergraduate and above coded as 0, 6, 9, 12, 15, 16, respectively. Marital status was a binary variable, with married coded as 1, and unmarried or widowed coded as 0. Place of residence was an ordinal variable, with rural areas coded as 1, towns coded as 2, and cities coded as 3. Personal annual income was a continuous variable. For descriptive analysis, income was divided into four levels. In the multiple linear regression model, the logarithm of personal annual income was taken.

### 3.3 Statistical analysis methods

This study conducted data analysis using Stata 17.0. Firstly, descriptive analysis was performed on the demographic variables of the sample. Secondly, independent sample T-tests and variance analysis were used to compare the differences in SH among different demographic groups. Thirdly, a correlation analysis was conducted on the core variables of this study. Fourthly, a multiple linear regression analysis method was applied to analyze the effect of the main independent variables on the SH of the older adult. The mathematical expression is shown in Equation 1:


(1) $$\begin{array}{l} SH=b_{0}+b_{1}\times FDU+b_{2}\times LMI+b_{3}\times DEI+\\ b_{4}\times \mathrm{SHDU}+b_{5}\times CV+e_{1} \end{array}$$

Where SH is subjective health, b1, b2, b3, and b4 are the regression coefficients of the independent variables FDU, LMI, DEI, and SHDU, respectively, CV is the control variable, and e1 is the error term. Finally, a chained mediation statistical method was used to analyze the influence mechanism of the three dimensions of DL on the SH of the older adult. The mathematical expressions are shown in Equations 2–5:


(2) $$LMI=b_{6}\times FDU+b_{7}\times CV+e_{2}$$

(3) $$DEI=b_{8}\times FDU+b_{9}\times CV+e_{3}$$

(4) $$\mathrm{SHDU}=b_{10}\times FDU+b_{11}\times LMI+b_{12}\times DEI+b_{13}\times CV+e_{4}$$

(5) $$\begin{array}{l} SH=b_{14}\times FDU+b_{15}\times LMI+b_{16}\times DEI+\\ b_{17}\times \mathrm{SHDU}+b_{18}\times CV+e_{5} \end{array}$$

Equations 2, 3 are the regression formulas for FDU on LMI and DEI for the older adult, respectively. Equation 4 is the regression formula for the use of FDU, LMI, and PDI on the SHDU by the older adult. Equation 5 is the regression formula for all independent and mediating variables on the SH of the older adult, where e2, e3, e4, and e5 are the error terms for each equation, respectively.

## 4 Result

### 4.1 Description of sample distribution

This study included a total of 2,086 valid cases. The demographic variables of the sample are presented in Table 1. In the sample, males (*n* = 1,092, 52.35%) were slightly more than females (n = 994, 47.65%). Most respondents were aged between 60 and 69 years (*n* = 1,631, 78.19%), followed by those aged 70–79 years (*n* = 386, 18.50%). Only a small portion of participants were aged 80–89 years (*n* = 61, 2.92%) or 90 years and above (*n* = 8, 0.38%). In terms of education level, secondary education (*n* = 879, 42.14%) was the most common, followed by those who had completed primary education (*n* = 519, 24.88%). A smaller portion of the older adult had completed high school education (*n* = 382, 18.31%) or held a bachelor’s degree or higher (*n* = 127, 6.09%), and only a few had not received any school education (*n* = 179, 8.58%). Most older adult lived in urban areas (*n* = 1,417, 67.91%), with fewer living in rural areas (*n* = 431, 20.67%) or towns (*n* = 238, 11.41%), which is basically consistent with the current urban–rural distribution of the population in China. Most participants were married (*n* = 1,731, 82.98%), with a smaller proportion being unmarried or widowed (*n* = 355, 17.02%). In terms of religious beliefs, the vast majority of participants reported having no religious beliefs (*n* = 1,943, 92.71%), with a few reporting having religious beliefs (*n* = 152, 7.29%). In terms of monthly income, those with an income exceeding 7,500 yuan last year (*n* = 767, 36.77%) accounted for the largest proportion, followed by those with an income between 4,001 yuan and 7,500 yuan (*n* = 588, 28.19%), and those with an income between 1,800 yuan and 4,000 yuan (*n* = 525, 25.17%). Those with an income below 1,800 yuan (*n* = 206, 9.88%) accounted for the smallest proportion.

**Table 1:** Socio-demographic characteristics of the respondents.

Total		Frequency	Percentage
Gender	Female	994	47.65%
	Male	1,092	52.35%
Age (years)	60–69	1,631	78.19%
	70–79	386	18.50%
	80–89	61	2.92%
	≥90	8	0.38%
Educational level	No schooling	179	8.58%
	Primary school	519	24.88%
	Middle school	879	42.14%
	High school	382	18.31%
	Bachelor’s degree and above	127	6.09%
Hometown	Rural	431	20.67%
	Towns	238	11.41%
	Cities	1,417	67.91%
Marriage	Unmarried or widowed	355	17.02%
	Married	1731	82.98%
Religious belief	No religious belief	1943	92.71%
	Religious belief	152	7.29%
Monthly income	<1800	206	9.88%
	1800–4,000	525	25.17%
	4,001–7,500	588	28.19%
	>7,500	767	36.77%

### 4.2 Description of older adult subjective health status

Table 2 presented the overall SH level of the older adult, as well as comparisons of SH among different demographic groups. The SH variable in this paper ranged from 1 to 5 points, with statistics showing that the average score of SH for the older adult was 3.406 ± 0.764, indicating that the overall SH score of the older adult was relatively high. This paper also compared the SH of different groups based on demographic variables. When the group category was two, an independent sample *T*-test was used, and when the group category was three or more, a one-way analysis of variance was used. In terms of gender, the SH of males (3.440 ± 0.745) was significantly higher than that of females (3.368 ± 0.782), with a significant difference between the two (*t* = −2.148, *p* < 0.05). In terms of age, the SH level of the older adult aged 60–69 (3.447 ± 0.751) was significantly higher than those aged 70–79 (3.254 ± 0.791), 80–89 (3.311 ± 0.807), and 90 and above (3.188 ± 0.594), with a significant difference (*F* = 7.240, *p* < 0.001). In terms of education level, the SH of the older adult with a bachelor’s degree or higher (3.592 ± 0.701) was significantly higher than that of the older adult with other education levels, with a statistically significant difference (*F* = 11.670, *p* < 0.001). In terms of residence, there was a significant difference in SH overall between rural (3.323 ± 0.826), town (3.433 ± 0.842), and city (3.427 ± 0.728) older adult people (*F* = 3.210, *p* < 0.05), with the SH level of town older adult people being higher than the other two groups. The SH score of married older adult people (3.424 ± 0.746) was significantly higher than that of unmarried or widowed older adult people (3.316 ± 0.839), with a significant difference (*t* = −2.436, p < 0.05). The SH of older adult people without religious beliefs (3.415 ± 0.756) was slightly higher than that of older adult people with religious beliefs (3.290 ± 0.844), but the difference was not significant (*t* = 1.932, *p* > 0.05). The SH status of older adult people with a monthly income exceeding ¥7,500 (3.486 ± 0.719) was significantly higher than that of older adult people with other income levels, with a statistically significant difference (*F* = 9.420, *p* < 0.001).

**Table 2:** Comparison of subjective health among different population groups.

		Score	T(F)	p
Total		3.406 ± 0.764		
Gender	Female	3.368 ± 0.782	−2.148	0.032^*^
	Male	3.440 ± 0.745		
Age(years)	60–69	3.447 ± 0.751	7.240	0.000^***^
	70–79	3.254 ± 0.791		
	80–89	3.311 ± 0.807		
	>90	3.188 ± 0.594		
Edu	No schooling	3.215 ± 1.018	11.670	0.000^***^
	Primary school	3.333 ± 0.634		
	Middle school	3.274 ± 0.792		
	High school	3.407 ± 0.708		
	Bachelor’s degree and above	3.592 ± 0.701		
Hometown	Rural	3.323 ± 0.826	3.210	0.041^*^
	Towns	3.433 ± 0.842		
	Cities	3.427 ± 0.728		
Marriage	Unmarried or widowed	3.316 ± 0.839	−2.436	0.015^*^
	Married	3.424 ± 0.746		
Religious belief	No religious belief	3.415 ± 0.756	1.932	0.053
	Religious belief	3.290 ± 0.844		
Monthly income	<1800	3.179 ± 0.862	9.420	0.000^***^
	1800–4,000	3.367 ± 0.863		
	4,001–7,500	3.414 ± 0.669		
	>7,500	3.486 ± 0.719		

^*^p < 0.05, ^***^p < 0.001.

### 4.3 Correlation analysis

Table 3 presents the correlation coefficients, significance levels, and effect sizes among the key variables. As shown in Table 3, there are significant positive correlations between SH and the frequency of digital information use (*r* = 0.116, *p* > 0.1), entertainment and leisure (*r* = 0.072, *p* < 0.01), life management (*r* = 0.120, *p* < 0.001), and the use of smart healthcare devices (*r* = 0.090, *p* < 0.001). Additionally, the frequency of digital information use is significantly positively correlated with entertainment and leisure (*r* = 0.125, *p* < 0.001), life management (*r* = 0.258, *p* < 0.01), and the use of smart healthcare devices (*r* = 0.164, *p* < 0.01). There is a positive correlation between entertainment and leisure and life management (*r* = 0.178, *p* < 0.001), but a negative correlation between entertainment and leisure and the use of smart healthcare devices (*r* = −0.048, *p* < 0.05). Lastly, there is a positive correlation between life management and the use of smart healthcare devices (*r* = 0.042, *p* < 0.1). Although the correlations between these variables have all reached a significant level, when considering the R-squared values as indicators of the effect size for the correlation coefficients, all effect sizes are found to be small. To explore the relationship between DL and the SH of older adults, further multivariate linear regression and mediation analyses May provide additional insights.

**Table 3:** Correlation analysis.

	1	2	3	4	5
1. Subjective health (SH)	1.000				
2. Frequency of Digital Usage (FDU)	0.116^***^ (0.013)	1.000			
3. Digital entertainment information (DEI)	0.072^**^ (0.005)	0.125^***^ (0.016)	1.000		
4. Life management information (LMI)	0.120^***^ (0.014)	0.258^**^ (0.067)	0.178^***^ (0.032)	1.000	
5. Smart healthcare device usage (SHDU)	0.090^***^ (0.008)	0.064^**^ (0.004)	−0.048^*^ (0.002)	0.042^+^ (0.002)	1.000

Effect size in parentheses ^+^*p* < 0.1, ^*^p < 0.05, ^**^p < 0.01, ^***^p < 0.001.

### 4.4 Regression analysis

Table 4 displays the regression analysis results of DL on the SH of older adults. Model 1 is the baseline model, which includes only the control variables. The statistical results show that gender is significantly and positively associated with SH (*b* = 0.077, *p* < 0.05), indicating that male older adult individuals are psychologically healthier than females. Age is significantly and negatively associated with SH (*b* = −0.019, *p* < 0.001), indicating that among the older adult, the younger they are, the better their SH. Education level is significantly and positively associated with SH (*b* = 0.026, *p* < 0.001), suggesting that the higher the educational level of the older adult, the better their SH. Monthly income is significantly and positively associated with SH (*b* = 0.080, *p* < 0.001), indicating that the higher the income of the older adult, the better their SH. The association between the remaining control variables and the SH of older adults is not statistically significant. Model 2 adds the FDU to Model 1. The coefficient for the FDU is significant (*b* = 0.053, *p* < 0.05), indicating that the higher the FDU among the older adult, the better their SH, thus confirming Hypothesis 1. Model 3 adds two variables related to the use of digital information content, namely DEI, and LMI, to Model 1. The statistical results show that the association between DEI and the SH of older adults is not significant (*b* = 0.268, *p* > 0.1), while LMI is significantly and positively associated with SH (*b* = 0.5, *p* < 0.001). This means that if older adult individuals use digital information mainly for life management, their SH will be better; conversely, if they use digital information mainly for entertainment and leisure, there is no significant alteration observed in their levels of SH. Model 4 adds the SHDU to Model 1. The statistical findings indicate a positive association between the use of smart health devices and SH (*b* = 0.864, *p* < 0.001), suggesting that the SHDU by the older adult contributes to their SH. Model 5 includes all control variables and the four variables encompassed by DL in the statistical analysis. The results show that LMI (*b* = 0.437, *p* < 0.01) and the SHDU (*b* = 0.842, *p* < 0.001) remain significantly positive, while the coefficients for the FDU and DEI are no longer significant.

**Table 4:** Regression analysis of the impact of digital literacy on subjective health.

	Model 1	Model 2	Model 3	Model 4	Model 5
Gender (Male = 1)	0.077^*^	0.078^*^	0.065^+^	0.082^*^	0.071^*^
	(0.036)	(0.036)	(0.036)	(0.036)	(0.036)
Age	−0.019^***^	−0.018^***^	−0.017^***^	−0.019^***^	−0.017^***^
	(0.003)	(0.004)	(0.003)	(0.003)	(0.004)
Education	0.026^***^	0.024^***^	0.024^***^	0.025^***^	0.022^***^
	(0.006)	(0.006)	(0.006)	(0.006)	(0.006)
Marriage (Married = 1)	0.018	0.026	0.027	0.017	0.030
	(0.049)	(0.049)	(0.049)	(0.049)	(0.049)
Hometown (Urban = 1)	−0.019	−0.025	−0.021	−0.022	−0.027
	(0.024)	(0.024)	(0.024)	(0.024)	(0.024)
Monthly income (ln)	0.080^***^	0.076^***^	0.073^***^	0.080^***^	0.070^***^
	(0.018)	(0.018)	(0.018)	(0.018)	(0.018)
Frequency of Digital Usage (FDU)		0.053^*^			0.032
		(0.024)			(0.024)
Digital entertainment information (DEI)			0.268		0.294
			(0.216)		(0.216)
Life management information (LMI)			0.500^***^		0.437^**^
			(0.146)		(0.148)
Smart healthcare device usage (SHDU)				0.864^***^	0.842^***^
				(0.222)	(0.222)
Intercept	0.555^*^	0.421	0.319	0.578^*^	0.256
	(0.273)	(0.280)	(0.299)	(0.272)	(0.302)
*N*	2086	2086	2086	2086	2086
*R^2^*	0.047	0.049	0.053	0.054	0.061
*ΔR^2^*	—	0.002	0.006	0.007	0.014
*p*	0.000	0.000	0.000	0.000	0.000

Standard errors in parentheses ^+^*p* < 0.1, ^*^p < 0.05, ^**^p < 0.01, ^***^p < 0.001.

### 4.5 Mediation analysis

**Figure 2:**
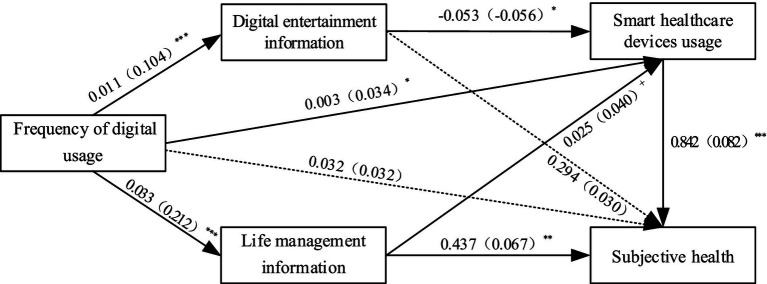
Path analysis diagram. ^*^p < 0.05, ^**^p < 0.01, ^***^p < 0.001, The data outside the parentheses denote unstandardized coefficients, while the data within the parentheses denote standardized coefficients.

Based on multiple linear regression analysis, this study used the SH of the older adult as the dependent variable, FDU as the independent variable, and digital information content and SHDU as mediating variables to form a chain mediation model. As shown in Figure 2, the path coefficients between the variables were presented, with the data outside the parentheses representing unstandardized path coefficients, and the data inside the parentheses representing standardized path coefficients. The FDU demonstrated positive associations with LMI (*b* = 0.033, *p* < 0.001), DEI (*b* = 0.011, *p* < 0.001), and SHDU (*b* = 0.003, *p* < 0.05), yet the direct effect of FDU on SH was not significant (*b* = 0.032, *p* > 0.1). DEI was negatively associated with SHDU (*b* = −0.053, *p* < 0.05), but its relationship with SH (*b* = 0.294, *p* > 0.1) was not significant, indicating no correlation between older adults’ online entertainment activities and their proficiency in SHDU, nor a significant relationship with their SH. LMI showed positive associations with SHDU (*b* = 0.025, *p* < 0.001) and SH (*b* = 0.437, *p* < 0.01), suggesting that older adults’ use of the Internet for life management could facilitate their technical skills in utilizing smart healthcare devices and was positively related to their SH. Lastly, SHDU was also positively associated with SH (*b* = 0.842, *p* < 0.001).

According to the mediation analysis results that were presented, the indirect effects of each mediating path were calculated as shown in Table 5. The path “FDU → DEI → SH” indicated no significant association between FDU and SH through DEI (*b* = 0.0031, *p* > 0.1), suggesting that older adults’ use of the Internet for entertainment and leisure did not have a clear relationship with their SH, and Hypothesis 2a was not supported. The path “FDU → LMI → SH” showed a positive association between FDU and SH through LMI (*b* = 0.0143, *p* < 0.01), indicating that the use of the Internet for life management skills could contribute to the improvement of SH status in older adults, and Hypothesis 2b was supported. The path “FDU → SHDU → SH” indicated a positive association between FDU and SH through SHDU (*b* = 0.0028, *p* < 0.05), suggesting that SHDU significantly enhanced the SH level of older adults, and Hypothesis 3 was supported. The mediating path “FDU → DEI → SHDU→ SH” also had a relatively small mediating coefficient (*b* = −0.0005, *p* < 0.05), indicating a certain negative association between FDU and SH of older adults through DEI and SHDU, and Hypothesis 4a was supported. The mediating path “FDU → LMI → SHDU→ SH “had a significant mediating coefficient (*b* = 0.0007, *p* < 0.1), indicating that FDU could positively associate with SH to some extent through LMI and SHDU, and Hypothesis 4b was supported.

**Table 5:** Mediation effect decomposition.

	*b*	SE	*p*	95% CI
FDU → DEI → SH	0.0031	0.0024	0.205	(−0.0016, 0.0078)
FDU → LMI → SH	0.0143	0.0046	0.002	(0.0055, 0.0230)
FDU → SHDU → SH	0.0028	0.0014	0.045	(0.0001, 0.0057)
FDU → DEI → SHDU→ SH	−0.0005	0.0002	0.049	(−0.0009, −0.0001)
FDU → LMI → SHDU→ SH	0.0007	0.0004	0.066	(−0.0001, 0.0014)

FDU, frequency of digital usage; DEI, digital entertainment information (DEI); LMI, life management information; SHDU, smart healthcare devices usage; SH, subjective health.

## 5 Discussion

The digital construction and aging population are rapidly advancing worldwide. Effectively utilizing digital empowerment for healthy aging could contribute to addressing the global issue of population aging. Previous research primarily focused on the relationship between FDU and SH. This paper expands the research question to explore the relationship between DL and SH.

Research has found that the frequency of using digital information had no direct association with the SH of the older adult, which differs from previous studies (39, 40). FDU is significantly correlated with DEI, LMI, as well as with SHDU. Furthermore, there is a significant association between the use of LMI and SHDU with the SH perceptions of older adults. Thus, FDU May indirectly relate to SH through the intermediary roles of LMI and SHDU. Older adult individuals can use digital health devices like smart wristbands to provide personalized health monitoring tools, remote medical services, and health management applications, enabling them to better control their health status and enhance their self-efficacy in health management (68), thus allowing them to make more proactive health decisions. Moreover, LMI and SHDU have a positive chained mediating effect on the SH of the older adult, while DEI and SHDU have a negative chained mediating effect on their SH. This suggests that the content of digital information and the usage skills of smart healthcare devices have diverse relations with the SH of the older adult. DEI did not help the older adult master smart healthcare devices, thereby indirectly negatively relationship with their SH. In summary, the above findings indicate that most dimensions of DL are significantly associated with the SH levels of the older adult. However, the associations between the acquisition of digital information, the selection of digital information, and digital usage skills with the SH of the older adult are not entirely consistent. Further illustrating that DL is a comprehensive concept comprising multiple dimensions. Its internal structure is not only multi-dimensional, but there is also a certain progressive relationship among the different dimensions of DL.

Previous studies rarely consider the content of digital information for the older adult as a major dimension of DL, and even fewer classify digital information content. This paper attempts to supplement these deficiencies in previous research. The distinctive findings of this study elucidate the variegated associations between diverse digital information content and the perceived health status of older adults. Specifically, digital information pertaining to life management is positively linked with the SH of the older adult, whereas the correlation between entertainment and leisure digital content and the SH of this demographic is not markedly evident. This study suggests that the observed phenomenon is associated with the fulfillment of the older adult’s life needs and that their self-assessment of health is linked to the extent to which their psychological needs are satisfied (85). LMI May help meet the life management needs of the older adult, help them better manage daily life, maintain healthy habits, plan retirement life, etc. (32, 60), enhance their autonomy, sense of competence, and seek support in life, thereby improving their quality of life, increasing life satisfaction, and enhancing their SH perception (61, 62).

Digital information for entertainment, such as online chatting, video streaming, and video games, exhibits multifaceted roles in meeting the needs of the older adult. On one hand, this type of digital information helps seniors pass the time in a relaxed atmosphere and, to some extent, aids in acquiring health knowledge through interactive entertainment, thereby enhancing their health awareness. It also contributes to improving their physical coordination and reaction speed. However, on the other hand, DEI can easily lead to addiction among the older adult, the experiences of diminished visual acuity, compromised sleep quality, and symptoms of anxiety May be correlated with internet addiction. Overall, Although digital information for entertainment and leisure has satisfied the recreational needs of the older adult to a certain extent, it also carries the potential to exert a negative influence on their SH. After the positive and negative aspects of this relationship neutralize each other, the association between digital information for entertainment and leisure and the SH of the older adult becomes non-significant. The “Uses and Gratifications” theory can elucidate the differentiated association between digital information content and the SH of the older adult. The older adult’s preferences for digital information content are driven by their specific needs, which are met to varying degrees by different types of digital content. Consequently, they tend to prioritize content that most effectively addresses their most significant needs. In contrast to entertainment and leisure digital information, health-related and other life management digital information is more intimately connected with the daily lives of the older adult. This category of information is better positioned to meet the older adult’s daily needs for health and wellness, suggesting a more substantial and evident connection between LMI and the SH perceptions of the older adult.

This study extends the investigation into the mechanisms linking DL with the SH of the older adult, analyzing the mechanisms underlying the differentiated relationships between various dimensions of DL and the SH of the older adult. Compared to previous research, The main innovation of this paper lies in the application of an expanded multidimensional DL framework to the older adult population, and the use of the “Uses and Gratifications” theory to elucidate the differences in the relationship between the digital information content selected under various needs and the SH of the older adult, thus expanding the explanatory scope of the “Uses and Gratifications” theory. This study enhances the understanding of healthy aging from a multidimensional DL perspective, providing significant policy implications for the ongoing digital construction and healthy aging initiatives across the globe. It is essential for digital infrastructure to deliver digital information that is accessible and suitable for the older adult, with a particular focus on supplying high-quality life management digital content. Additionally, establishing avenues to assist the older adult in mastering the use of digital health devices is crucial. These efforts collectively aim to elevate the older adult’s comprehensive DL, thereby empowering them to better leverage DL for healthy aging.

This study acknowledges several limitations. The use of secondary data has constrained the operationalization of variables, particularly in the comprehensive assessment of DL and SH among the older adult. The measurement of variables was limited by the number of items and the binary approach used to assess the engagement with digital information content and the utilization of smart healthcare devices. This has led to relatively small effect sizes among the primary variables in this study, a lower *R*-squared value for the regression model, and minimal variation in the *R*-squared values across different models. Additionally, the absence of longitudinal data limits the inference of causality, which should be addressed in future research. Future research should adopt a more comprehensive and multidimensional approach to assessing SH and DL. This should involve enhancing the granularity and precision of variable measurement and data collection to more effectively investigate the explanatory power of DL on SH. Future inquiries should also analyze the interplay between DL and social factors, such as social capital, to comprehensively elucidate the mechanisms by which digital social dynamics influence the SH of the older adult following their integration into DL frameworks.

## 6 Conclusion

The findings of this study demonstrate a significant positive association between overall DL and SH among the older adult. The frequency of digital information use, LMI, and the SHDU are directly or indirectly linked to the SH of the older adult. Moreover, LMI and smart health devices exhibit a positive mediating effect between the frequency of digital information use and SH. In contrast, the chained relationship between entertainment and leisure digital information and smart health devices has a negative mediating effect between the frequency of digital information use and SH, while the direct effect of DEI on SH is not significant. These insights underscore the positive impact of enhancing DL among the older adult on their SH. Consequently, it is essential to provide the older adult with more life management digital information and to assist them in mastering the use of digital health devices, thereby better harnessing the empowering potential of digital construction for the health of the older adult.

## Data Availability

Publicly available datasets were analyzed in this study. This data can be found here: Restrictions apply to the availability of these data. Data were obtained from the National Survey Research Center at the Renmin University of China and are available at http://class.ruc.edu.cn/, with the permission of the National Survey Research Center at Renmin University of China.
