# Supplementary material for: Enhancement or suppression: a double-edged sword? Differential association of digital literacy with subjective health of older adult—evidence from China
Source: Front Public Health. 2024 Sep 20;12:1395162. doi: 10.3389/fpubh.2024.1395162 (PMC11449883; doi:10.3389/fpubh.2024.1395162)
Supplement: Supplementary file 1 [file Data_Sheet_1.docx]

**Appendix 1. Formal Questionnaire**

1. How do you rate your current physical health status?

(1) Very unhealthy (2) Unhealthy (3) Average (4) Healthy (5) Very healthy

2. Compared to your peers, how do you feel about your health status?

(1) Much worse (2) Somewhat worse (3) About the same (4) Somewhat better (5) Much better

3. Do you use the internet?

(1) Never use the internet (2) A few times a year (3) At least once a month (4) At least once a week (5) Every day

4. How frequently have you used the internet in the past three months?

(1) Never (2) Rarely (3) Sometimes (4) Often (5) Always

5. How frequently have you used mobile phone to customize messages in the past three months?

(1) Never (2) Rarely (3) Sometimes (4) Often (5) Always

6. What do you generally do when you go online?

| **No.** | **Activity** | **Yes (1)** | **No (0)** |
| --- | --- | --- | --- |
| 1 | Text chatting |  |  |
| 2 | Reading news |  |  |
| 3 | Listening to music, watching videos |  |  |
| 4 | Shopping |  |  |
| 5 | Transportation and travel |  |  |
| 6 | Health management |  |  |
| 7 | Investment and financial management |  |  |

7. Have you ever used the following smart devices? (Multiple choices allowed)

| **No.** | **Activity** | **Yes (1)** | **No (0)** |
| --- | --- | --- | --- |
| 1 | Smart wheelchair |  |  |
| 2 | Smart wristband |  |  |
| 3 | Smart sleep monitor |  |  |
